# Comparison of Inflammatory Mediators in Patients With Atrial Fibrillation Using Warfarin or Rivaroxaban

**DOI:** 10.3389/fcvm.2020.00114

**Published:** 2020-07-24

**Authors:** Gabriela Lopes Martins, Rita Carolina Figueiredo Duarte, Érica Leandro Marciano Vieira, Natalia Pessoa Rocha, Estêvão Lanna Figueiredo, Francisco Rezende Silveira, José Raymundo Sollero Caiaffa, Rodrigo Pinheiro Lanna, Maria das Graças Carvalho, András Palotás, Cláudia Natália Ferreira, Helton José Reis

**Affiliations:** ^1^Neurofar Laboratory, Departamento de Farmacologia, ICB, Universidade Federal de Minas Gerais, Belo Horizonte, Brazil; ^2^Department of Neurology, The University of Texas Health Science Center at Houston, Houston, TX, United States; ^3^Hospital Lifecenter, Belo Horizonte, Brazil; ^4^Hospital Semper, Belo Horizonte, Brazil; ^5^Centro de Especialidades Médicas Ipsemg, Belo Horizonte, Brazil; ^6^Asklepios-Med, Szeged, Hungary; ^7^Kazan Federal University, Kazan, Russia

**Keywords:** atrial fibrillation, inflammation, warfarin, rivaroxaban, cytokines, chemokines

## Abstract

**Background:** Atrial fibrillation (AF) is the most common arrhythmia associated with high risk of venous thromboembolism. Inflammatory mechanisms may be involved in the pathophysiology of AF and in the AF-related thrombogenesis, and patients with AF might benefit from the use of anticoagulants with anti-inflammatory properties. However, the evidence is still scarce, and it points out the need of trials seeking to investigate the levels of inflammatory mediators in patients with AF under different anticoagulant therapies. Therefore, this study was designed to define whether patients with AF treated either with an activated coagulation factor X (FXa) inhibitor (rivaroxaban) or with a vitamin K inhibitor (warfarin) present changes in peripheral levels of inflammatory mediators, mainly cytokines and chemokines.

**Methods:** A total of 127 subjects were included in this study, divided into three groups: patients with non-valvular atrial fibrillation (NVAF) using warfarin (*N* = 42), patients with NVAF using rivaroxaban (*N* = 29), and controls (*N* = 56). Plasma levels of inflammatory mediators were quantified by immunoassays.

**Results:** Patients with AF (both warfarin and rivaroxaban groups) presented increased levels of inflammatory cytokines in comparison with controls. The use of rivaroxaban was associated with decreased levels of inflammatory cytokines in comparison with warfarin. On the other hand, patients with AF using rivaroxaban presented increased levels of the chemokines (MCP-1 in comparison with warfarin users; MIG and IP-10 in comparison with controls).

**Conclusions:** AF is associated with an inflammatory profile that was less pronounced in patients on rivaroxaban in comparison with warfarin users. Further studies are necessary to assess the clinical implications of our results and whether patients with AF would benefit from rivaroxaban anti-inflammatory effects.

## Introduction

Atrial fibrillation (AF) is the most frequent cardiac arrhythmia in clinical practice. AF prevalence is estimated to be about 1–2% in the general population, increasing significantly with age (1). AF is associated with a considerable risk of mortality and morbidity due to venous thromboembolism (VTE). People with AF have increased risk of mortality compared to those without it, as demonstrated by the Framingham Heart Study (2). Noteworthy, AF remained significantly associated with an increased risk of death even after controlling for cardiovascular comorbidities (2, 3). AF is also associated with an estimated incremental cost of US$18,601 in hospital and clinical care in the year after diagnosis, even after accounting for age, time period, and comorbidities (4).

For clinical risk stratification, the CHA_2_DS_2_-VASc^1^ score has been widely accepted for predicting VTE in patients with AF and used as a surrogate for starting anticoagulant therapy based on the individuals' assessment (5–8). Warfarin is an inhibitor of vitamin K activity, being highly effective in preventing stroke and VTE in patients with AF and is the most used oral anticoagulant in clinical practice (9). However, due to the narrow therapeutic index and interactions with several drugs and foods, warfarin use requires frequent coagulation monitoring and dose adjustment (10). As an attempt to mitigate this problem, new oral anticoagulants have been developed, including rivaroxaban, which is a direct inhibitor of the activated coagulation factor X (FXa). Rivaroxaban has been proved to be non-inferior to warfarin in preventing stroke and VTE, with the great advantage of providing a more consistent and predictable pharmacological profile (11). FXa may play an important role in the activation of inflammatory responses (12), thus yielding another potential advantage of anti-FXa drugs, i.e., their anti-inflammatory effects (13, 14).

Inflammatory responses, as evidenced by increased circulating levels of inflammatory mediators such as C-reactive protein (CRP), promote the persistence of AF (15, 16). In addition, the number of T lymphocytes and monocytes/macrophages is increased in the atrial myocardium of patients with AF (17). Regarding thrombus formation, higher plasma levels of CRP and interleukin (IL)-6 have been independently associated with stroke risk (18–20). Taken together, these data demonstrate that patients with AF might benefit from the use of anticoagulants with anti-inflammatory properties. Accordingly, a recent study has demonstrated that the treatment with rivaroxaban (43–98 days, depending on cardioconversion) was associated with reductions in both CRP and IL-6 in comparison with baseline levels (21). However, the evidence is still scarce, and it points out the need for trials seeking to investigate the levels of inflammatory mediators in patients with AF under different anticoagulant therapies. Therefore, this study was designed to define whether patients with AF treated with rivaroxaban (FXa inhibitor) or warfarin (vitamin K inhibitor) present changes in peripheral levels of inflammatory mediators, mainly cytokines and chemokines. We hypothesize that the use of rivaroxaban is associated with a decrease in the levels of inflammatory mediators.

## Methods

### Study Population

A total of 127 participants were enrolled in the study, 71 patients with non-valvular atrial fibrillation (NVAF) and 56 controls with comparable age and sex distribution. Participants were recruited from the outpatient clinics of the Hospitals Lifecenter, Semper, and Ipsemg (Belo Horizonte, Minas Gerais, Brazil). Patients were selected if they (i) presented with a history of AF, whose diagnosis was confirmed by electrocardiography, and (ii) had a prescription for chronic oral anticoagulation (CHA2DS2-VASC ≥ 2). Patients with AF were further categorized according to anticoagulant treatment: *N* = 42 were on warfarin with international normalized ratio (INR) between 2.00 and 3.00, and *N* = 29 were on rivaroxaban treatment, at a dose of 20 mg once daily. Controls were recruited from the local community and consist of a group of individuals with no previous diagnosis of AF or use of any anticoagulant drug.

Subjects were excluded if they have used any antiplatelet agent, non-steroidal anti-inflammatory drugs, heparin, hormone replacement therapy, antifibrinolytics, amiodarone, verapamil, quinidine, azole antifungals, and ritonavir in the 4 weeks prior to the study. In addition, participants with current diagnosis of alcohol use disorder; chronic kidney disease (creatinine clearance <30 ml/min); severe dyslipidemia; acquired or hereditary bleeding disorders; liver disease; thyroid disease; infectious, inflammatory, autoimmune, and malignant diseases; pregnancy; puerperium; and breast-feeding were excluded from this study.

The present study was approved by the Research Ethics Committees of *Universidade Federal de Minas Gerais* (UFMG–CAAE: 12603413.0.0000.5149), Lifecenter, Semper, and Ipsemg Hospitals and was performed in accordance with the principles provided in the Declaration of Helsinki. All participants received information about the research, and read and signed a written informed consent prior to any study procedures.

### Biological Samples

Peripheral blood samples were drawn by venipuncture into tubes containing ethylenediamine tetraacetic acid (EDTA) and into tubes without anticoagulant in the Hospitals Lifecenter, Semper, and Ipsemg. Plasma and serum samples were obtained by centrifuging blood at 1,100 *g* for 15 min, at 25°C, within 4 h of collection. The samples were labeled and stored in aliquots at −80°C until analysis.

### Laboratory Characterization

Serum samples were used for the assessment of the following biochemical markers: CRP, total cholesterol, high-density lipoprotein (HDL), low-density lipoprotein (LDL), triglycerides, aspartate transaminase (AST), alanine aminotransferase (ALT), gamma-glutamyl transferase (GGT), creatinine, and uric acid, using a Vitros 250 system (Johnson & Johnson®) in the Laboratory of *Hospital Risoleta Tolentino Neves* (Belo Horizonte, Minas Gerais, Brazil). In addition, white blood cell (WBC) count was assessed in whole blood samples by using the Coulter T-890® hematological analyzer in the *Laboratório de Hematologia Cl*í*nica, Faculdade de Farmácia*, UFMG.

### Analysis of Inflammatory Mediators

Plasma samples were used for the assessment of inflammatory markers by Cytometric Bead Array (CBA, BD Bioscience, San Diego, CA, USA), using the following kits: Cytokines Th1/Th2 [IL-2, IL-4, IL-10, tumor necrosis factor (TNF), and interferon-gamma (IFN-γ)], Chemokines [CCL5/regulated on activation, normal T cell expressed and secreted (RANTES), CXCL9/monokine induced by interferon-gamma (MIG), CCL2/monocyte chemoattractant protein (MCP)-1, CXCL10/interferon-gamma-induced protein (IP)-10], and human transforming growth factor-beta (TGF-β)-1 Single Plex Flex Set. In addition, multiplex immunoassay (Merck Millipore, Billerica, MA, USA) was used to assess the levels of cardiovascular disease-related proteins, using the Human Cardiovascular Disease-Panel II kit [a disintegrin and metalloproteinase with a thrombospondin type 1 motif, member 13 (ADAMTS13), growth differentiation factor (GDF)-15, myoglobin, soluble intercellular adhesion molecule (sICAM)-1, p-selectin, neutrophil gelatinase-associated lipocalin (lipocalin-2/NGAL), soluble vascular cell adhesion protein (sVCAM)-1, and serum amyloid A (SAA)].

### Statistical Analyses

Associations between categorical variables were assessed by the Pearson chi-square test. The Shapiro–Wilk normality test was used to assess whether or not the continuous variables follow a normal distribution. Three groups (warfarin vs. rivaroxaban vs. controls) were compared using the one-way ANOVA or the Kruskal–Wallis test, when data were determined to follow or not a normal distribution. Significant results for three-group comparisons were followed by *post hoc* analyses (Tukey's or Dunn's pairwise comparisons, in case of ANOVA or Kruskal–Wallis test, respectively). Significant values have been adjusted by the Bonferroni correction for multiple tests. All statistical tests were two-tailed and a significance level of *p* < 0.05 was set. Statistical analyses were performed using SPSS software version 25.0 (SPSS Inc., Chicago, IL, USA).

## Results

### Demographic, Clinical, and Laboratory Characteristics

Demographic, clinical, and laboratory characteristics of participants enrolled in this study are shown in Table 1. There were no differences between groups regarding age, sex, diagnosis of type II diabetes mellitus. The frequency of hypertension was higher among patients with AF (both warfarin and rivaroxaban groups) in comparison with controls. The percentage of patients using statins was higher in the warfarin group, in comparison with controls and with patients on rivaroxaban. There was no significant difference between patients on warfarin or rivaroxaban regarding the CHA_2_DS_2_-VASc score. Only 6 out of the 127 participants reported tobacco use (3/56 controls, 1/42 patients with AF on warfarin, and 2/29 patients on rivaroxaban). Due to the low expected frequencies, chi-square analysis for smoking status was not appropriate.

**Table 1:** Demographic, clinical, and laboratory characteristics of participants.

**Parameters**	**Controls (*N* = 56)**	**Warfarin (*N* = 42)**	**Rivaroxaban (*N* = 29)**	***P***
Age, in years	72 ± 7	71 ± 7	73 ± 10	0.344^1^
Sex				
Female, *N* (%)	34 (60.7%)	20 (47.6%)	13 (44.8%)	0.273^2^
Male, *N* (%)	22 (39.3%)	22 (52.4%)	16 (55.2%)	
Hypertension, *N* (%)	32 (57.1%)^a^	40 (95.2%)^b^	29 (100%)^2^	**<0.001^2^**
Diabetes Mellitus 2, *N* (%)	7 (12.5%)	14 (33.3%)	9 (31.0%)	0.094^2^
Statins, *N* (%)	22 (39.3%)^a^	26 (61.9%)^b^	11 (37.9%)^a, b^	**0.046^2^**
CHA_2_DS_2_-VASc	-	3 (2.5–4)	4 (3–4)	0.776^3^
Total cholesterol (mg/dl)	189 ± 38^a^	177 ± 41^a, b^	167 ± 34^b^	**0.044^1^**
LDL (mg/dl)	102 ± 30	89 ± 35	87 ± 28	0.058^1^
HDL (mg/dl)	51 (43–66.25)	55 (45.25–66.25)	47 (40–64.5)	0.418^4^
Triglycerides (mg/dl)	136.5 (103–182.25)	125.5 (96.5–232)	123 (88–190.5)	0.520^4^
ALT (U/L)	24.5 (18.75–29.25)	27 (22.25–37.75)	23 (16–30)	0.093^4^
AST (U/L)	26 (22.75–30)^a^	30.5 (25–39.25)^b^	25 (22.5–32.5)^a, b^	**0.014^4^**
GGT (U/L)	24 (20.5–35)^a^	46 (30.25–69.25)^b^	42 (23.5–65)^b^	**<0.001^4^**
Creatinine (mg/dl)	0.9 (0.8–1.1)^a^	1.1 (1–1.3)^b^	1.1 (0.9–1.35)^b^	**0.001^4^**
Uric acid (mg/dl)	5.45 (4.58–6.28)^a^	6.3 (5.3–8.15)^b^	6.4 (5.7–7.3)^b^	**0.001^4^**
WBC	5647 ± 1649^a, b^	5005 ± 1348^a^	6152 ± 1440^b^	**0.020**^1^
CRP (mg/L)	8 (6–10.5)^a^	5 (5–7)^b^	7 (5–9)^a, b^	**0.004^4^**

1 *ANOVA (values expressed as mean and standard deviation)*.

2 *Pearson chi-square test (values expressed as absolute and relative frequency)*.

3 *Mann–Whitney test (values expressed as median and 25th−75th percentiles)*.

4 *Kruskal–Wallis test (values expressed as median and 25th−75th percentiles)*.

*Significant results for three-group comparisons were followed by post hoc analyses (Tukey's or Dunn's pairwise comparisons, in case of ANOVA or Kruskal–Wallis test, respectively). Significant values have been adjusted by the Bonferroni correction for multiple tests and are indicated by different letters*.

*CHA_2_DS_2_-VASc, Congestive heart failure, Hypertension, Age ≥75 (doubled), Diabetes, Stroke (doubled), Vascular disease, Age 65–74, Sex category (female), LDL, low-density lipoprotein, HDL, high-density lipoprotein, ALT, alanine aminotransferase, AST, aspartate transaminase, GGT, gamma-glutamyl transferase, WBC, white blood cells, CRP, C-reactive protein. Bold values indicate statistically significant differences between groups, with a significance level of p < 0.05*.

Table 1 shows the mixed array of biochemical markers in the three groups of participants. It also shows that patients on warfarin presented lower levels of CRP compared to controls. In addition, patients on rivaroxaban showed higher values in WBC count compared to the warfarin group. Of note, these parameters were determined in order to exclude patients presenting with acute inflammatory response and/or severe dyslipidemia or liver disease.

### Evaluation of Inflammatory Mediators

**Figure 1:**
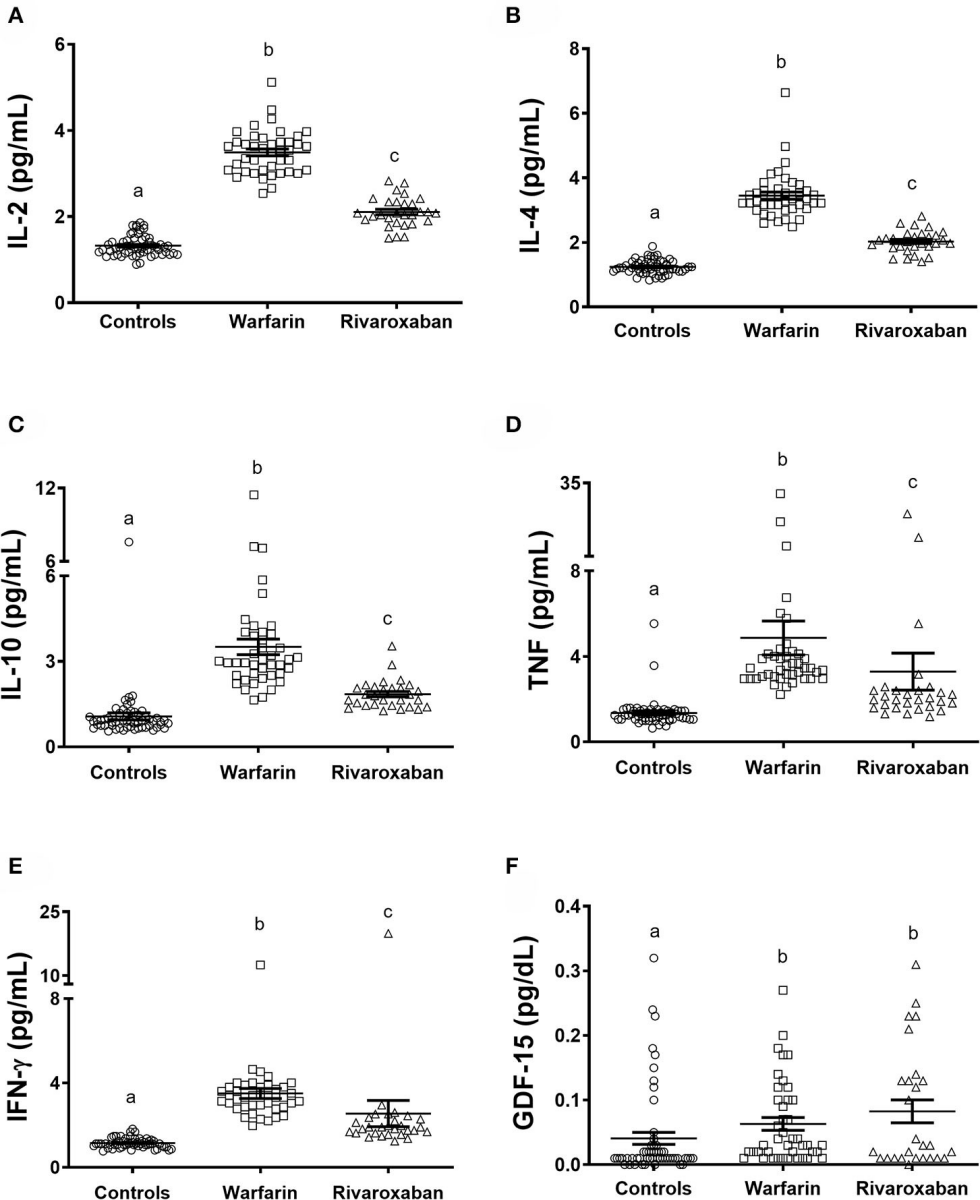
Plasma levels of inflammatory cytokines IL-2 **(A)**, IL-4 **(B)**, IL-10 **(C)**, TNF **(D)**, IFN-γ **(E)**, and GDF-15 **(F)**, comparing control (*N* = 56), warfarin (*N* = 42), and rivaroxaban (*N* = 29) groups. The dosage of IL-2, IL-4, IL-10, TNF, and IFN-γ was performed by Cytometric Bead Array; GDF-15, by Multiplex Immunoassay. The groups were compared by the Kruskal–Wallis test followed by Dunn's multiple comparison test. Significant values have been adjusted by the Bonferroni correction for multiple tests and are indicated by different letters. The horizontal bars show the mean and the standard error of the mean. IL, interleukin; TNF, tumor necrosis factor; IFN-γ, interferon-gamma; GDF-15, growth differentiation factor-15.

Increased levels of inflammatory cytokines were found in individuals with AF in comparison with controls. Patients on warfarin, compared with those on rivaroxaban and controls, presented with increased plasma levels of IL-2, IL-4, IL-10, TNF, and IFN-γ. Moreover, increased levels of these markers were found in the rivaroxaban group in comparison with controls (Figures 1A–E, respectively). Regarding the cardiovascular disease-related proteins, patients on warfarin and rivaroxaban presented similar levels of GDF-15 and both groups higher than controls (Figure 1F).

**Figure 2:**
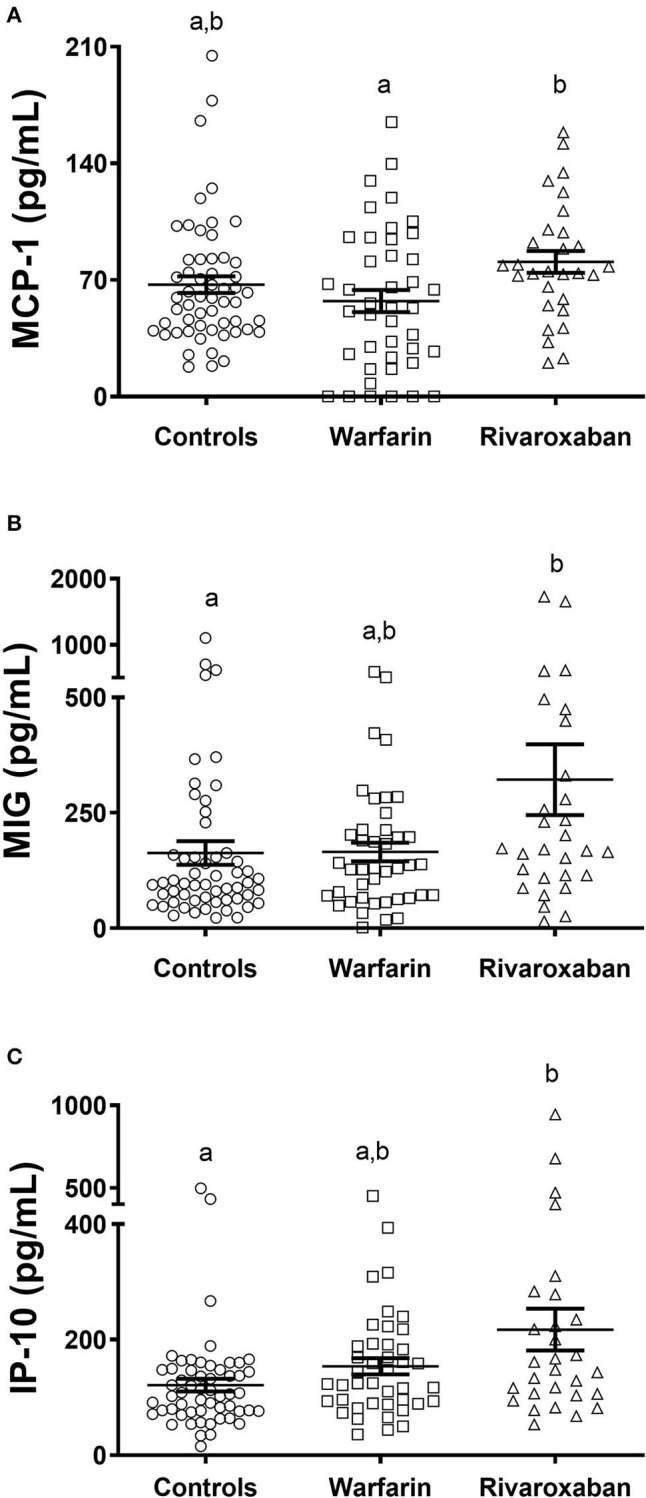
Plasma levels of inflammatory chemokines MCP-1 **(A)**, MIG **(B)**, and IP-10 **(C)**, comparing control (*N* = 56), warfarin (*N* = 42), and rivaroxaban (*N* = 29) groups. The dosage of these markers was performed by Cytometric Bead Array. The groups were compared by the Kruskal–Wallis test followed by Dunn's multiple comparison test. Significant values have been adjusted by the Bonferroni correction for multiple tests and are indicated by different letters. The horizontal bars show the mean and the standard error of the mean. MCP-1, monocyte chemoattractant protein-1; MIG, monokine induced by interferon-gamma; IP-10, interferon-gamma-induced protein.

On the other hand, the rivaroxaban group had significantly higher levels of MCP-1 in comparison with the warfarin group (Figure 2A). In addition, patients on rivaroxaban presented increased levels of MIG and IP-10 in comparison with controls (Figures 2B,C).

For the other analyzed parameters (RANTES, TGF-β, ADAMTS13, myoglobin, sICAM-1, p-selectin, lipocalin-2/NGAL, sVCAM-1, and SAA), no significant differences were found when comparing the three groups (data not shown).

## Discussion

In the current study, we evaluated plasma levels of inflammatory mediators in patients with AF on two different anticoagulant therapies, i.e., warfarin and rivaroxaban. Previous studies have demonstrated that in addition to the anticoagulant effects, FXa inhibitors such as rivaroxaban have anti-inflammatory properties (12, 13). We then hypothesized that rivaroxaban would be associated with decreased levels of inflammatory cytokines. We found that patients with AF (both warfarin and rivaroxaban groups) presented increased levels of inflammatory cytokines in comparison with controls. Corroborating our hypothesis, the use of rivaroxaban was associated with decreased levels of inflammatory cytokines in comparison with warfarin (Figure 1). Interestingly, patients with AF using rivaroxaban presented increased levels of the chemokines [MCP-1 in comparison with warfarin users; MIG and IP-10 in comparison with controls (Figure 2)].

Our results are in line with findings from previous studies, which reported increased plasma levels of inflammatory cytokines in patients with AF (22–25). Inflammatory mechanisms have been associated with the pathogenesis and maintenance of AF, as well as in the AF-related thrombogenesis (19). Furthermore, comorbidities shared by AF and increased risk of stroke, such as hypertension and diabetes, are also associated with inflammation (26). Thus, it is reasonable to hypothesize that patients with AF would benefit from drugs exerting anti-inflammatory properties.

IL-2 is a pro-inflammatory cytokine produced by T-helper type 1 (Th1) lymphocytes, which can activate T and natural killer (NK) cells and plays a crucial role in the regulation of T cells (25, 27). In a previous study, this cytokine was considered an independent predictive factor for recurrence of AF, once elevated plasma levels of IL-2 were detected in patients with AF who presented recurrence of AF after catheter ablation therapy, in comparison with control subjects in sinus rhythm (28). IL-4, in turn, is a cytokine expressed and produced by activated T-helper type 2 (Th2) lymphocytes and basophils, which has regulatory actions on allergic responses, as well as anti-tumor and anti-inflammatory effects (22). This mediator was considered as a predictive marker of the incidence of AF since high levels of this cytokine were found in patients admitted to the hospital with AF in comparison with controls (22). IL-10, an anti-inflammatory cytokine produced by monocytes and Th2 lymphocytes, and IFN-γ, an immunomodulatory cytokine produced by NK cells and Th1 lymphocytes, were associated with the development of AF after coronary artery bypass grafting (24). Finally, TNF, a pro-inflammatory cytokine synthesized by monocytes, macrophages, and Th1 lymphocytes, has been described as a predictive factor for the development of ischemic stroke in patients with chronic NVAF (29). Our results showed that the increase in levels of these cytokines in patients with AF was blunted in the rivaroxaban group, thus concluding that the use of this drug may be beneficial over warfarin in the context of inflammatory mechanisms.

The anti-inflammatory effects of FXa inhibitors were further demonstrated by a study in which the 6-months treatment with rivaroxaban or apixaban reduced the values of pentraxin-related protein (PTX-3), a protein produced primarily by macrophages and endothelial vasculature cells in response to early pro-inflammatory signals. The authors then suggested that PTX-3 would respond rapidly to endothelial changes in the left atrium and vessels, serving as a useful marker to determine the anti-inflammatory effect of FXa inhibitors (13). The anti-inflammatory activity of rivaroxaban was found to be associated with the inhibition of FXa-activated inflammatory response. When human atrial tissue slices were exposed to FXa, the expression of ICAM-1 and IL-8 increased. The combination of rapid pacing and FXa (mimicking AF) has promoted the significant up-regulation of the protease-activated receptor (PAR-1), PAR-2, ICAM-1, and IL-8. Rivaroxaban prevented the up-regulation of PARs, ICAM-1, and IL-8. In summary, these results indicate that FXa mediates inflammatory signaling in atrial tissue, possibly by the activation of protease-activated receptors (12). Considering the evidence that FXa can bind to PARs, activating them (30), the potential anti-inflammatory effect of rivaroxaban may be because of a direct FXa inhibition.

Contrary to the cytokines, the levels of chemokines were higher in rivaroxaban users in comparison with warfarin (MCP-1) and controls (MIG and IP-10, Figure 2). Chemokines are chemoattractant cytokines that play a vital role in cell migration to the damaged tissue, needed for after-injury remodeling. MCP-1 is a chemokine produced by several cell types, either constitutively or after induction by oxidative stress, cytokines, or growth factors. MCP-1 acts as a potent chemotactic factor for monocytes/macrophages, and it has been associated with cardiac fibrosis and remodeling in ischemic cardiomyopathy, and after myocardial infarction (31). This may be due to its effects on the recruitment and activation of mononuclear cells and fibroblast progenitors, with subsequent release of collagen, fibronectin, and other extracellular matrix components (32). The inhibition of MCP-1 in infarcted mice has been shown to impair the myocardial repair process after injury (33). MCP-1 appears to be involved not only in cell recruitment but also in the modulation of cardiac homeostasis after injury (34). Regarding IP-10, this is a chemokine with pro-inflammatory, anti-angiogenic, and anti-fibrotic properties. It was demonstrated that IP-10 is up-regulated in myocardial infarction in mice, acting as an inhibitor of growth factor-induced fibroblast migration, which protects the heart from excessive fibrotic remodeling (35, 36). In the same way, MIG is a pro-inflammatory chemokine induced by IFN-γ, with proven anti-fibrotic effects in *in vitro* liver cells (37). Therefore, the increase in chemokine levels observed in the rivaroxaban group can be regarded as beneficial, as chemokines are vital for tissue remodeling and resolution of inflammatory responses. Further investigations are required to evaluate if the use of rivaroxaban is directly associated with an increase of plasmatic levels of these chemokines in patients with AF and the related clinical implications.

## Study Limitations

The results of the current study should be interpreted taking into account its limitations. First, our results cannot be extrapolated to patients with AF using other oral anticoagulants. Second, the use of other drugs (such as antihypertensive and oral hypoglycemic agents) was very common in our sample, and it cannot be terminated for the sake of scientific research for obvious ethical considerations. Therefore, the observed findings might also be influenced by their ongoing treatments. Third, the fact the data were not normally distributed (even after attempts to data transformation) prevented the use of more sophisticated analyses. Lastly, the sample size and the cross-sectional design hampered any statements about the potential anti-inflammatory effect of rivaroxaban and the clinical implications of our results. Prospective studies with larger sample size are needed to validate the current findings.

## Conclusion

We demonstrated that patients with AF presented with increased levels of inflammatory cytokines. This profile was different when we compared patients treated with warfarin or rivaroxaban. It is possible that rivaroxaban has a potential anti-inflammatory effect. However, the cross-sectional nature of our study prevents this assumption. Further prospective studies involving a greater number of participants are required to confirm the effects of oral anticoagulants in relation to the inflammatory response in individuals with AF. Altogether, our results point to new perspectives for the treatment of patients AF with oral anticoagulants with anti-inflammatory effects.

## Data Availability Statement

The raw data supporting the conclusions of this article will be made available by the authors, without undue reservation.

## Ethics Statement

The studies involving human participants were reviewed and approved by Ethics Committee from UFMG. The patients/participants provided their written informed consent to participate in this study.

## Author Contributions

GM, CF, and HR worked on the conception and organization of the research project. Material preparation, data collection, and analysis were performed by GM, CF, RD, ÉV, NR, EF, FS, JC, and RL. GM wrote the first draft of the manuscript. RD, ÉV, NR, EF, FS, JC, RL, MC, AP, CF, and HR reviewed the manuscript. All authors read and approved the final version of the manuscript.

## Conflict of Interest

The authors declare that the research was conducted in the absence of any commercial or financial relationships that could be construed as a potential conflict of interest.

## Footnotes

^1^Congestive heart failure, Hypertension, Age ≥75 (doubled), Diabetes mellitus, prior Stroke/transient ischemic attack/thromboembolism (doubled), Vascular disease, Age 65–74, Sex category (female).
